# Is International or Asian Criteria-based Body Mass Index Associated with Maternal Anaemia, Low Birthweight, and Preterm Births among Thai Population?—An Observational Study

**DOI:** 10.3329/jhpn.v29i3.7869

**Published:** 2011-06

**Authors:** Tippawan Liabsuetrakul

**Affiliations:** Epidemiology Unit, Faculty of Medicine, Prince of Songkla University, Hat Yai, Songkhla, 90112, Thailand; ^*^Members of the Working Group include: Tippawan Liabsuetrakul, Department of Obstetrics and Gynecology, Faculty of Medicine, Prince of Songkla University, Hat Yai, Songkhla, Thailand; Pravit Chaikongkeit, Division of Obstetrics and Gynecology, Health Promotion Center, 12 Yala, Yala, Thailand; Suwannee Korviwattanagarn, Division of Obstetrics and Gynecology, Health Promotion Center, 12 Yala, Yala, Thailand; Chulaphorn Petrueng, Yala Hospital, Yala, Thailand; Surangkana Chaiya, Yala Hospital, Yala, Thailand; Chintana Hanvattanakul, Bannangsata Hospital, Yala, Thailand; Pisun Kongkitkul, Division of Obstetrics and Gynecology, Pattani Hospital, Pattani, Thailand; Chamaiporn Sinthuuthai, Pattani Hospital, Pattani, Thailand; Noree Kalong, Yarang Hospital, Amphur Yarang, Pattani, Thailand; Darunee Ongsawang, Bajoe Hospital, Amphur Bajoe, Narathiwat, Thailand; Sirinart Ungsathapornpon, Su-ngai Kolok Hospital, Su-ngai Kolok, Narathiwat, Thailand; Apiradee Ameeroh, Su-ngai Kolok Hospital, Su-ngai Kolok, Narathiwat, Thailand; Pornwilai Bavonnarongdet, Thepa Hospital, Amphur Thepa, Songkhla, Thailand; and Arom Buadung, Ranod Hospital, Amphur Ranod, Songkhla, Thailand

**Keywords:** Anaemia, Body mass index, Observational studies, Pregnancy, Thailand

## Abstract

An observational study was conducted in the four southernmost provinces of Thailand aiming at determining the effect of international or Asian criteria-based body mass index (BMI) in predicting maternal anaemia, low birthweight (LBW), and preterm births among pregnant Thai women and the change in haemoglobin (Hb) level during pregnancy. Maternal anaemia was defined as a haemoglobin (Hb) level of <11 g/dL. Anaemia was detected in 27.4% and 26.9% of 1,192 pregnant women at their first prenatal visit and the third trimester respectively. The proportions of overweight and obese women according to the Asian criteria-based pre-pregnancy BMI were higher than the international criteria-based BMI (22.4% and 10.1% vs 15.5% and 3.4% respectively). No significant difference between pre-pregnancy BMI and pregnancy BMI at the first prenatal visit was demonstrated (mean±standard deviation=21.8±4.0 vs 22.8±4.1). Underweight women had a significantly higher prevalence of maternal anaemia, LBW, and preterm birth compared to women with normal weight. Overweight and obese women at pre-pregnancy by the Asian criteria-based BMI had a lower prevalence of anaemia. The Hb levels did not change significantly over time. In addition to BMI, maternal age, parity, and late prenatal visit were independently associated with maternal anaemia, low birthweight, and preterm birth. Underweight pregnant women classified by international or Asian criteria-based BMI increased the risk of maternal anaemia, low birthweight, and preterm birth.

## INTRODUCTION

Pregnant women are vulnerable and prone to developing physiological and pathological anaemia. Maternal anaemia is a burden throughout the world, especially in developing countries (1). Maternal anaemia is defined as a haemoglobin (Hb) level of <11g/dL, or haematocrit (Hct) of <33% in all trimesters of pregnancy as defined by the World Health Organization (WHO) (2). A high prevalence of anaemia increases the risk of maternal death in Africa, Asia, and Latin America (1). Anaemia in pregnancy is also associated with malnourishment and low socioeconomic conditions (3,4). Nutritional status can be measured using various parameters, such as weight, height, body mass index (BMI), triceps skinfold, or mid-upper arm circumference. However, BMI is most commonly used in the research field in developing countries. Low maternal BMI has a strong relationship with maternal anaemia (4-8).

The nutritional status of pregnant women is not only related to anaemia but also to the poor pregnancy outcomes in both developing and developed countries. Low maternal BMI results in an increased incidence of newborns with low birthweight (LBW), an Apgar score of less than 5 at one minute and perinatal mortality in Sudan (5). Underweight, pregnant women in North India had a higher occurrence of anaemia; in contrast, obese pregnant women tend to develop diabetes mellitus, pregnancy-induced hypertension, and are more likely to give birth by caesarean section due to foetal distress and cepahlopelvic disproportion (9). Likewise, underweight women had a significant higher rate of newborns with foetal anaemia, LBW, and preterm delivery but a lower rate of late booking for prenatal care, gestational diabetes mellitus, pre-eclampsia, and postpartum haemorrhage in England (10).

The effects of pre-pregnancy BMI, BMI at the first prenatal visit, or gestational weight gain associated with adverse pregnancy outcomes have been previously reported (11-14). The implication of BMI at the first prenatal visit or gestational weight gain is dependent on the gestational age of pregnancy at the first prenatal visit and gestational age at birth. Similarly, the pre-pregnancy BMI may be affected by recall bias if it is self-reported. In addition, the BMI cut-points for classifying body-weight categories have been found to vary in previous studies.

Underweight has been defined for BMI of less than the 5th percentile for age—18.5 or 19.8. Overweight has been defined for BMI of the ≥85^th^ percentile for age or ≥23, ≥25, or ≥30 (7,9,15). According to the WHO, the four BMI cut-point categories were defined as follows: underweight (BMI <18.5), normal weight (BMI 18.5-24.9), overweight (BMI 25-29.9), and obese (BMI ≥30) (9-11). Recently, the WHO experts addressed the recommended cut-points for BMI categories in Asian populations as follows: <18.5, 18.5-23, 23-27.5, and ≥27.5 for underweight, normal weight, overweight and obese respectively (16).

There is a lack of evidence showing the association between Asian criteria-based BMI and maternal anaemia, LBW, and preterm delivery among the Asian population. Therefore, this study aimed at determining the risk of anaemia, LBW, and preterm delivery according to the international and Asian criteria-based BMI during pre-pregnancy and pregnancy at the first prenatal visit and the changes in Hb and Hct during pregnancy.

## MATERIALS AND METHODS

### Study design

The study was a part of the cohort project on epidemiology of infestation of soil-transmitted helminths investigating the effects of treatment, the prevalence of anaemia, and nutritional status in pregnancy. The Institute Ethics Committee of the Faculty of Medicine, Prince of Songkla University (reference no. 49/370-004) approved the study. Information on epidemiology of infestation of soil-transmitted helminths and the effect of its treatment was published in 2009 (17).

### Study settings and subjects

The present study was conducted in nine hospitals located in the four southernmost provinces of Thailand, namely Songkhla, Pattani, Yala, and Narathiwat, during March 2006–June 2007. All pregnant women who lived in the service areas of the participating hospitals and attended for their first prenatal visit were included. Women who had a gestational age of more than 32 weeks due to a limited time for follow-up before delivery or those who had a history of antihelminthic drug allergies were excluded. To detect a 10% difference of anaemia prevalence between underweight women and normal-weight women using a ratio of 1 to 3, at least 231 underweight women and 558 normal-weight women were required.

### Data-collection

#### Preparatory phase

The quality assurance of body-weight digital machines for pregnant women (Glass Digital Scale, Tesco Stores Ltd., UK) and for newborns (1583 Baby Scale, Tanita Corporation of America, Inc.) and also the complete blood count machines, routinely used in the hospitals (Hmx Hematology Analyzer, Backman Coulter; ABX Pentra 60, Horiba or MEN-6318k&NEK8222k, Nihon Kohden) were checked. Nine body-weight digital machines for pregnant women were similarly accurate with coefficient of variations of weight ranging from 0.10% to 0.16%. The standardization of complete blood count machine was performed by comparing the results of Hb levels with the well-known standard values of Hb levels. The finding showed that the reliability of tested Hb results was good with coefficient of variations ranging from 1.4% to 1.7%.

#### Data-collection phase

All eligible women were approached consecutively. Those agreeing to participate and give written informed consent were asked to provide baseline information on demographic, socioeconomic and obstetric factors (age, religion, education, occupation with low or high exposure of soil-contact, fami-ly income, place of shower, place of defaecation, infestation of soil-transmitted helminths (STHs), parity and gestational age at the first prenatal visit), and daily food intake. The first, second and third trimesters of pregnancy were classified using gestational age as follows: <14, 14-28, and >28 weeks respectively. The recommendation for daily food intake for pregnant women published in a maternal and childcare handbook provided by the Ministry of Public Health, Thailand, was considered the guidance of six food-groups (rice, vegetable, meat, fruit, fat, and milk) and amount of foods required. Photographs of various foods were used for facilitating the accuracy of the estimated portion-size of food intake. Body-weight of eligible women shortly before pregnancy was self-reported by an interview and recorded as their pre-pregnancy weight.

At enrollment, body-weight and height of all the recruited women were measured during the first prenatal visit and then recorded as their pregnancy-weight. Blood was drawn and analyzed for Hb and Hct. All the women were followed with routine, standard prenatal care until delivery. Information on maternal pregnancy-weight, Hb and Hct at each trimester and delivery (if available), LBW, and preterm birth were also recorded.

According to the policy of routine prenatal care, all pregnant women were assessed for Hb or Hct at their first prenatal visit and again at the third trimester. Those who were not anaemic received one tablet of ferrous sulphate (60 mg of elemental iron) daily at 14-20 weeks of pregnancy until delivery. Those who were anaemic were treated with 2 or 3 tablets daily until anaemia was resolved. Stool examination for infestation of STHs was not a routine policy in pregnancy but it was used for the study through the well-standardized, modified Kato-Katz techniques (18).

#### Definitions of variables

Maternal anaemia in this study was defined as Hb level of <11 g/dL or Hct of <33%. The degrees of severity of anaemic were classified as mild (Hb 9.0-10.9 g/dL), moderate (Hb 7.0-8.9 g/dL) and severe anaemia (Hb <7 g/dL) (19). BMI was calculated as body-weight in kg divided by height in metre squared (kg/m^2^). Pre-pregnancy weight and pregnancy weight at the first prenatal visit were used for calculating pre-pregnancy and pregnancy BMI. Asian criteria-based BMI was used as follows: <18.5 for underweight, 18.5-22.9 for normal-weight, 23.0-27.5 for overweight, and >27.5 for obese women. International criteria-based BMI was used as follows: <18.5 for underweight, 18.5-24.9 for normal-weight, 25.0-29.9 for overweight, and ≥30 for obese women (16). LBW referred to a foetal birthweight of less than 2,500 g, and preterm birth was defined as a foetus born before 37 completed weeks.

### Data-processing and analysis

Data were entered in the EpiData software (version 3.1) (The EpiData Association, Denmark, 2004) and analyzed using the R software (version 2.7.0) (the R Foundation for Statistical Computing, Austria, 2008). The effect of international and Asian criteria-based pre-pregnancy and pregnancy BMI and other independent variables of mothers on anaemic status at the first prenatal visit, LBW and preterm birth were explored by univariate analysis using the chi-square test. Variables which showed a p value of less than 0.2 by univairate analysis were added in the first model of multiple logistic regression, and then the significant variables were kept in the final model using a backward-stepwise method if a p value was less than 0.05 by the likelihood ratio test. The change of Hb or Hct at each trimester was analyzed using the linear mixed-effects modelling technique fit by maximum likelihood.

## RESULTS

A diagram of the study participants and the three main outcomes is shown in the figure. The sociodemographic and nutritional status of the participants is shown in Table 1 and Table 2. The age of the 1,192 eligible women ranged from 13 to 46 years [mean±standard deviation (SD)=27.1±6.1]. Three-fourths had attended primary or secondary school, and a half had a family income of US$ 150-300 per month. Twenty percent of the participants had not drunk milk in the one month before interview. The proportions of overweight and obese women based on the Asian criteria-based pre-pregnancy BMI were higher than the international criteria-based BMI (22.4% and 10.1% vs 15.5% and 3.4% respectively). No significant difference was demonstrated between pre-pregnancy BMI and pregnancy BMI at the first prenatal visit (mean±SD=21.8±4.0 vs 22.8±4.1).

**Table 1.** Sociodemographic characteristics of study participants at the first prenatal visit

Variable	No.	%
Age (years)		
<20	133	11.2
20-34	916	76.9
≥35	142	11.9
Parity		
0	474	39.8
1-3	623	52.2
≥4	95	8.0
Trimester at first visit		
First	485	40.7
Second	676	56.7
Third	31	2.6
Religion		
Non-Muslim	311	26.1
Muslim	881	73.9
Level of education		
<Secondary school	432	36.2
Secondary or diploma	584	49.0
≥Bachelor	176	14.8
Occupation*		
Low exposure of soil-contact	723	60.7
High exposure of soil-contact	469	39.3
Family income (US$) per month		
≤300	880	73.8
>300	312	26.2
Place of shower		
Inside house	967	81.1
Outside house	225	18.9
Place of defaecation		
Inside latrine	1166	97.8
Outside latrine	26	2.2
Infestation of soil-transmitted helminths		
No	897	75.3
Yes	195	16.4
Unknown	100	8.4

*Low exposure of soil-contact: merchant, government and state enterprise employee and student; High exposure of soil-contact: gardener, farmer, fisherman, labourer, and housewife

**Table 2.** Nutritional status of study participants at the first prenatal visit

Variable	No.	%
Types and amount of daily food intake		
Rice (ladles)		
0	2	0.2
1-3	192	16.1
4-6	629	52.8
7-9	335	28.1
>9	34	2.0
Meat (tablespoons)		
0	7	0.6
1-4	168	14.1
5-8	561	47.1
9-12	394	33.1
>12	62	5.2
Vegetables (ladles)		
0	36	3.0
1-2	310	26.0
3-4	471	39.5
5-6	338	28.4
>6	36	3.1
Fruits (units)		
0	34	2.9
1-2	253	21.2
3-4	461	38.7
5-6	330	27.7
>6	114	9.6
Lipid (teaspoons)		
0	8	0.7
1-3	417	35.0
4-6	754	63.3
>6	13	1.1
Milk (glass/250 mL)		
0	243	20.4
1-3	898	75.3
4-6	47	3.9
>6	4	0.3
Pre-pregnancy BMI		
International criteria-based		
Underweight	231	19.4
Normal	720	60.4
Overweight	185	15.5
Obese	40	3.4
Unknown	16	1.3
Variable	No.	%
Asian criteria-based		
Underweight	231	19.4
Normal	558	46.8
Overweight	267	22.4
Obese	120	10.1
Unknown	16	1.3
Pregnancy BMI		
International criteria-based		
Underweight	125	10.5
Normal	748	62.8
Overweight	244	20.5
Obese	69	5.8
Unknown	6	0.5
Asian criteria-based		
Underweight	125	10.5
Normal	578	48.5
Overweight	326	27.3
Obese	156	13.2
Unknown	6	0.5

BMI=Body mass index

Table 3 shows the mean and SD of Hb, Hct, and BMI at each trimester and at birth and pre-pregnancy BMI. The mean values of Hb and Hct were the lowest at the second trimester but they did not significantly change over time. The mean BMI significantly (p<0.001) increased at each trimester. The prevalence of anaemia was 12.7%, 37.5%, 26.9%, and 18.3% at the first, second, and third trimester and at delivery respectively. Overall, the prevalence of anaemia at the first prenatal visit was 27.4%, and at the third trimester, it was 26.9%. Of 327 anaemic pregnant women at the first prenatal visit, the prevalence of mild, moderate, and severe anaemia was 91.7%, 2.3%, and 0% respectively.

**Table 3.** Levels of Hb, Hct, and BMI at each period

Variable	Pre-pregnancy	First trimester	Second trimester	Third trimester	At birth	p value*
Gestational age						
Mean (SD)	-	9.4(2.3)	20.3(4.3)	33.3(2.4)	38.8(2.0)	<0.001
Hb						
Mean (SD)	-	12.2(1.1)	11.3(1.2)	11.7(1.5)	12.2(1.4)	0.40
Hct						
Mean (SD)	-	36.7(3.8)	33.9(3.6)	35.2(4.2)	35.1(3.8)	0.90
BMI						
Mean (SD)	21.8(4.0)	22.4(4.1)	23.5(4.1)	25.8(3.8)	26.7(4.0)	<0.001

*Linear mixed-effects model fit by maximum likelihood;

BMI=Body mass index;

Hb=Haemoglobin;

Hct=Haematocrit;

SD=Standard deviation

The fitted logistic regression models predicting the odds of maternal anaemia, LBW, and preterm birth adjusted by other significant variables are presented in Table 4, 5, and 6 respectively. Both international and Asian criteria-based BMI at pre-pregnancy and pregnancy at the first prenatal visit were associated with the risk of maternal anaemia at the first prenatal visit after adjusting for maternal age, religion, occupation, parity, and place of shower. The underweight women identified by pre-pregnancy BMI had an increased risk of anaemia; in contrast, the overweight and obese women had a lower risk both by pre-pregnancy and pregnancy BMI. Maternal age of <20 years, non-muslim, low soil-contamination, grand multiparity, late trimester of the first prenatal visit, and shower outside a house were independently associated factors for maternal anaemia (Table 4).

**Table 4.** Final logistic regression model of anaemia at the first prenatal visit with international and Asian criteria-based BMI at pre-pregnancy and pregnancy at the first prenatal visit

Variable	Risk of anaemia in pregnancy by odds ratio (95% confidence interval)							
	Pre-pregnancy				Pregnancy			
	International criteria		Asian criteria		International criteria		Asian criteria	
	Crude	Adjusted	Crude	Adjusted	Crude	Adjusted	Crude	Adjusted
BMI								
Normal	-	-	-	-	-	-	-	-
Underweight	1.6 (1.2-2.2)	1.6 (1.2-2.3)**	1.4 (1.0-2.0)	1.5 (1.0-2.1)*	0.8 (0.5-1.2)	0.9 (0.6-1.5)	0.8 (0.5-1.2)	0.9 (0.6-1.5)
Overweight	1.0 (0.7-1.4)	0.9 (0.6-1.4)	0.7 (0.5-0.9)	0.7 (0.5-0.9)*	0.6 (0.4-0.9)	0.6 (0.4-0.8)**	0.9 (0.7-1.2)	0.8 (0.6-1.2)
Obese	0.3 (0.1-0.9)	0.3 (0.1-0.9)*	0.6 (0.4-1.0)	0.5 (0.3-0.9)*	0.5 (0.3-1.0)	0.4 (0.2-0.8)**	0.5 (0.3-0.8)	0.4 (0.2-0.6)***
Missing data	2.2 (0.8-6.1)	2.2 (0.8-6.6)	2.0 (0.7-5.4)	2.0 (0.7-5.8)	0.5 (0.05-3.9)	0.4 (0.04-4.3)	0.5 (0.05-4.0)	0.4 (0.04-4.4)
Age (years)								
20-34		-		-		-		-
<20		1.8 (1.2-2.8)**		1.9 (1.2-2.8)**		1.8 (1.2-2.8)**		1.8 (1.2-2.8)**
≥35		1.1 (0.7-1.7)		1.1 (0.7-1.8)		1.0 (0.7-1.7)		1.0 (0.6-1.6)
Religion								
Non-Muslim		-		-		-		-
Muslim		0.6 (0.5-0.9)**		0.6 (0.5-0.9)**		0.6 (0.5-0.9)**		0.7 (0.5-0.9)**
Occupation								
Low exposure of soil-contact				-		-		-
High exposure of soil-contact		0.7 (0.5-0.9)*		0.8 (0.6-1.0)*		0.8 (0.6-1.0)		0.7 (0.6-0.98)*
Parity								
0		-		-		-		-
1-3		1.0 (0.7-1.4)		1.1 (0.8-1.5)		1.0 (0.8-1.4)		1.0 (0.8-1.4)
≥4		1.9 (1.1-3.4)*		2.0 (1.1-3.6)*		2.1 (1.2-3.8)*		2.1 (1.2-3.8)*
Trimester at the first prenatal visit								
First		-		-		-		-
Second		3.8 (2.7-5.2)***		3.8 (2.7-5.2)***		3.9 (2.8-5.3)***		3.9 (2.8-5.3)***
Third		6.1 (2.8-13.4)***		6.2 (2.8-13.7)***		7.5 (3.4-16.6)***		7.4 (3.4-16.4)***
Place of shower								
Inside		-		-		-		-
Outside		1.6 (1.2-2.3)*		1.7 (1.2-2.3)**		1.7 (1.2-2.3)**		1.6 (1.2-2.3)**

*p<0.05;

**p<0.01;

***p<0.001;

BMI=Body mass index

Women classified as underweight using the international or Asian criteria-based pre-pregnancy BMI were not at a significantly higher risk of LBW. However, a higher risk of LBW was found for underweight women using pregnancy BMI at the first prenatal visit. Obese women, as defined by Asian criteria-based pre-pregnancy and pregnancy BMI, had a lower risk of LBW. Nulliparous women had a higher risk of LBW (Table 5). Underweight, defined by both international and Asian criteria-based pre-pregnancy BMI, was not associated with preterm birth. However, using the pregnancy BMI criteria, the risk of preterm birth was significantly higher among underweight women and in women aged <20 or ≥35 years (Table 6).

**Table 5.** Final logistic regression model of LBW with international and Asian criteria-based BMI at pre-pregnancy and pregnancy at the first prenatal visit

Variable	Risk of LBW in pregnancy by odds ratio (95% confidence interval)							
	Pre-pregnancy				Pregnancy			
	International criteria		Asian criteria		International criteria		Asian criteria	
	Crude	Adjusted	Crude	Adjusted	Crude	Adjusted	Crude	Adjusted
BMI								
Normal	-	-	-	-	-	-	-	-
Underweight	1.5 (1.0-2.5)	1.4 (0.9-2.2)	1.3 (0.8-2.1)	1.2 (0.8-2.0)	2.2 (1.3-3.7)*	2.0 (1.2-3.5)*	2.0 (1.1-3.4)*	1.9 (1.1-3.3)*
Overweight	0.7 (0.4-1.3)	0.8 (0.4-1.5)	0.6 (0.3-1.1)	0.7 (0.4-1.3)	0.6 (0.3-1.1)	0.7 (0.4-1.3)	0.6 (0.3-1.0)	0.7 (0.4-1.2)
Obese	0.3 (0.04-2.1)	0.3 (0.05-2.5)	0.2 (0.04-0.7)	0.2 (0.04-0.7)*	0.2 (0.02-1.1)	0.2 (0.02-1.1)	0.4 (0.2-0.9)	0.4 (0.2-0.99)*
Parity								
0		-		-		-		-
1-3		0.4 (0.3-0.7)***		0.5 (0.3-0.7)***		0.4 (0.3-0.7)***		0.5 (0.3-0.7)**
≥4		0.7 (0.3-1.6)		0.8 (0.4-1.9)		0.8 (0.4-1.8)		0.8 (0.4-1.9)
Amount of daily lipid intake (teaspoons)								
0		-		-		-		-
1-3		0.2 (0.04-1.2)		0.2 (0.03-1.0)*		0.2 (0.03-1.1)		0.2 (0.04-1.1)
4-6		0.3 (0.05-1.5)		0.2 (0.04-1.3)		0.3 (0.05-1.4)		0.3 (0.05-1.5)
>6		1.0 (0.1-8.1)		0.8 (0.1-7.1)		0.9 (0.1-8.1)		1.0 (0.1-8.7)

*p<0.05;

**p<0.01;

***p<0.001;

BMI=Body mass index;

LBM=Low birthweight

**Table 6.** Final logistic regression model of preterm birth with international and Asian criteria-based BMI at pre-pregnancy and pregnancy at the first prenatal visit

	Risk of preterm birth in pregnancy by odds ratio (95% confidence interval)							
Variable	Pre-pregnancy				Pregnancy			
	International criteria		Asian criteria		International criteria		Asian criteria	
	Crude	Adjusted	Crude	Adjusted	Crude	Adjusted	Crude	Adjusted
BMI								
Normal	-	-	-	-	-	-	-	-
Underweight	1.3 (0.8-2.1)	1.3 (0.8-2.1)	1.3 (0.8-2.2)	1.3 (0.7-2.2)	1.9 (1.1-3.3)*	1.9 (1.0-3.4)*	2.0 (1.1-3.7)*	2.0 (1.1-3.8)*
Overweight	0.9 (0.5-1.6)	0.8 (0.4-1.6)	1.0 (0.6-1.7)	1.0 (0.6-1.8)	1.0 (0.6-1.7)	1.0 (0.5-1.7)	1.2 (0.7-2.0)	1.2 (0.7-2.0)
Obese	1.6 (0.6-4.3)	1.6 (0.6-4.2)	1.0 (0.5-2.1)	1.0 (0.5-2.0)	0.9 (0.3-2.3)	0.8 (0.3-2.0)	1.0 (0.5-2.0)	1.0 (0.5-2.0)
Age (years)								
20-34		-		-		-		-
<20		2.1 (1.2-3.7)*		2.1 (1.2-3.6)*		2.0 (1.2-3.6)*		2.1 (1.2-3.6)*
≥35		1.9 (1.0-3.4)*		1.9 (1.0-3.4)*		1.9 (1.1-3.5)*		1.9 (1.0-3.4)*
Infestation of STHs								
No		-		-		-		-
Yes		1.2 (0.7-2.1)		1.2 (0.7-2.1)		1.2 (0.7-2.2)		1.2 (0.7-2.1)
Unknown		2.5 (1.4-4.6)**		2.6 (1.4-4.7)**		2.6 (1.4-4.8)**		2.6 (1.4-4.7)**

*p<0.05;

**p<0.01;

***p<0.001;

BMI=Body mass index;

TSHs=Soil-transmitted helminths

## DISCUSSION

Both pre-pregnancy and pregnancy BMI, using the international or Asian criteria-based classifications, were associated with maternal anaemia at the first prenatal visit and LBW and preterm birth in pregnant Thai women. The risk of maternal anaemia was higher in underweight women and lower in overweight and obese women based on pre-pregnancy BMI. From our results, underweight by pregnancy BMI could be used for predicting LBW and preterm birth. Maternal age, religion, occupation, parity, and late prenatal visit were also independently contributable factors.

The criteria for diagnosing anaemia of the WHO and Centers of Disease Control and Prevention (CDC) are minimally different, especially in the second trimester of pregnancy (3,5,20). The prevalence of anaemia in our study was high (27.4%) and was the highest at the second trimester; however, most women were only mildly anaemic—a similar finding reported from a study in Pakistan (3). However, results of two studies in Nepal showed that 32.4% of women who were anaemic had moderate and severe anaemia (21), and the prevalence of anaemia was 33.7%, 32%, and 31.7% at the first, second, and third trimester respectively (22). Moderate and severe anaemia were detected more in underweight women (17% and 4% respectively) than in overweight women (9% and 1% respectively) in a study in India (4). According to the population-based Swedish Medical Birth Register, women with high Hb levels at the first prenatal visit were more likely to have a high BMI (7). This is in accordance with a study in Nepal, results of which showed that the lowest prevalence of anaemia was found in women with the highest BMI, and Hct increased with an increase in BMI (22). A high prevalence of moderate and severe anaemia was found among pregnant tribal adolescents in one study in India, and most (83%) of these were malnourished (23).

Currently, the WHO recommends using both international and Asian criteria-based BMI. The cut-off value for classifying underweight is the same but for overweight and obese women, the values are different (16). The application of BMI in health research has been varied for pre-pregnancy and pregnancy (11-14). Likewise, different cut-off values of BMI have been published (13,14,24). Results of our literature search showed that the majority of studies used the international classification of BMI. The results of our study supported that either Asian or international criteria-based BMI is suitable for predicting maternal anaemia, LBW, and preterm birth. Low BMI increased not only the risk of anaemia but also the risk of other poor pregnancy outcomes as reported from both developing and developed countries (5,10,12,14,24-27). Although overweight and obese women had a lower risk of anaemia and LBW in our study, a higher prevalence of postpartum anaemia was detected in the obese women due to the increase of blood loss at delivery (28).

Other studies found that maternal anaemia was higher in pregnant women who were underweight (3,4), younger (22), had a low standard of living (4,22), and who drank alcohol (4) but was lower for those who had higher education (4,22) and were overweight (4). In our study, maternal age of <20 years was a strong predictor of anaemia and preterm birth, which was also supported by previous studies (8,22,23,26,27). Delay in the first prenatal visit after the first trimester, especially at the third trimester, resulted in a higher risk of detection of anaemia, which was similar to a report in north Thailand, although the CDC diagnostic criteria of anaemia was applied (20). Increased risk of LBW in nulliparous women was also confirmed by evidence from a systematic review (29). The association between anaemia and infestation of STHs in pregnancy could not be identified. Likewise, results of a study by Nurdiati *et al.* in Indonesia revealed that hookworm was not significantly associated with either Hb or ferritin levels (30). In contrast, showering outside the house, a significant factor of infestation of STHs found in our previous study (17), also showed to be a significant factor for the prevalence of anaemia. This might be because of other unidentified factors which were related to showering outside the house. Results of a study showed that LBW and preterm delivery increased significantly in women with severe anaemia at the first trimester (21). This finding could not be identified in our study, possibly because the majority of women had mild anaemia.

### Limitations

Our study had several limitations. First, pre-pregnancy weight was measured by self-reported interview which may be affected by recall bias. However, all the women were interviewed at their first prenatal visit; thus, recall bias was minimized. Second, the follow-up of Hb and Hct during pregnancy was a routine practice, and approximately half of the women's Hct was measured at the third trimester or at delivery. Third, the daily food intake was measured by the prenatal-care nurses with a modified food-frequency questionnaire, not a 24-hour recall food-frequency questionnaire. However, we applied the photos of local Thai foods based on guidance of the Ministry of Public Health and photos of portion-size to correctly estimate food intake in routine prenatal practice. Fourth, the compliance of iron supplementation was not recorded because the counting of iron tablets is not routinely performed in prenatal clinics. However, we considered maternal anaemia at the first prenatal visit so the compliance of iron intake was not affected. Finally, missing data on infestation of STH was a significant factor for preterm birth, for which the reason could not be explained.

### Conclusions

The results of the present study confirm that both Asian and international BMI classifications are appropriate for pregnant women of Thailand. Pre-pregnancy BMI is a predictor for maternal anaemia at the first prenatal visit, and pregnancy BMI at the first prenatal visit is a predictor of LBW and preterm birth. In addition, maternal age, parity, and late prenatal visit were independently associated risks of maternal anaemia. Further studies in other pregnant Asian women are needed to confirm the generalizability of the association and consequently its clinical application.

## ACKNOWLEDGEMENTS

Funding support for the study was provided by the Institute of Research and Development for Health of Southern Thailand. The authors appreciate the cooperation of various organizations, hospitals, and health personnel, particularly the Faculty of Medicine, Prince of Songkla University, the Chief Medical Officers of Songkhla, Pattani, Yala, and Narathiwat Provincial Health Offices, Health Center 12 Yala, and the Hospital Directors and prenatal-care team of the participating hospitals.
